# The top 100 most-cited articles on adult spinal deformity: The most popular topics are still sagittal plane parameters and complications

**DOI:** 10.3389/fsurg.2022.961582

**Published:** 2023-01-06

**Authors:** Fu-Sheng Liu, Lin-Xia Deng, Fu-Bing Liu, Qian-Shi Zhang, Xiao-Bin Wang, Jing Li

**Affiliations:** ^1^ Department of Spine Surgery Spinal Deformity Center, The Second Xiangya Hospital, Central South University, Changsha, China; ^2^ Department of Pediatrics, The Third Xiangya Hospital, Central South University, Changsha, China

**Keywords:** bibliometric, most-cited articles, adult spinal deformity, trends, visualization analysis

## Abstract

**Purpose:**

This study aimed to summarize the characteristics of the 100 most-cited articles on adult spinal deformity (ASD) and to analyze past and current research hotspots and trends.

**Methods:**

Literature searches (from inception to 28 April 2022) using Web of Science databases were conducted to identify ASD-related articles. The top 100 most-cited articles were collected for further analysis. Meanwhile, author keywords from articles published in the last 5 years were selected for further analysis.

**Results:**

The top 100 most-cited articles on ASD were selected from 3,354 papers. The publication year ranged from 1979 to 2017, and all papers were written in English. The citation count among them ranged from 100 to 1,145, and the mean citation number was 215.2. The foremost productive first author was Schwab F. University of Washington had the largest number of publications. The United States of America had the largest number of published articles (*n* = 84) in this field. *Spine* was the most popular journal. Complications were the most studied themes. The visualization analysis of author keywords from the literature in the recent 5 years showed that complications, sagittal plane parameters, and surgical techniques are still the research hotspots, and minimally invasive surgery will continue to develop rapidly.

**Conclusion:**

Based on a comparative analysis of the results of bibliometric and visualization, complications and sagittal plane parameters are still the major topics of research at present and even later, and minimally invasive surgery has a growth trend in this field of ASD.

## Introduction

Adult spinal deformity (ASD) is a complex rigid three-dimensional spinal deformity characterized by back pain, symptoms of nerve compression, malalignment, and decreased quality of life (1–3). The question of whether the optimal treatment of ASD is conservative management or operative treatment remains controversial. Compared with non-surgical treatment, correction surgery has shown favorable results for ASD, such as restoring alignment, reducing pain, and improving health-related quality of life (HRQOL) (4–6); however, these benefits are offset by high incidences of complications, the complexity of surgical techniques, and the uncertainty of long-term outcomes (7, 8). As people live longer lives and suffer from spinal degeneration, the prevalence of ASD grows and public attention is on the rise. Despite the high frequency of ASD, an understanding of the underlying mechanisms remains incomplete. Therefore, an in-depth study on the research status and hotspots is meaningful and necessary.

Summarizing the previous studies is of great practical significance in a specific research field. Bibliometrics refers to the quantitative analysis of a given topic, based on the existing published literature, using mathematical and statistical methods to quickly understand the hotspots, trends, author cooperation networks, institutional cooperation networks, article relevance analysis, and so on (9–11). Meanwhile, we can understand it intuitively by combining it with visualization software, such as VOSviewer and CiteSpace.

Although bibliometric analysis has been widely used in the field of medicine (11, 12), there is currently no detailed analysis of ASD. This paper collected and selected the 100 most influential articles in ASD and compared them with the literature published in the recent 5 years in order to better understand the cooperative network, academic community, current status, and research hotspots, while also discussing and summarizing the research trend in this field.

## Materials and methods

### Study design

In this study, the records of literature on ASD were analyzed using a bibliometric methodology.

### Search strategy

Electronic searches of the Web of Science database (including the Web of Science Core Collection, KCL-Korean Journal Database, MEDLINE, Russian Science Citation Index, and SciELO Citation Index) were performed from inception to 28 April 2022 (a time span from 1950 to 2022), to identify eligible studies. The terms used for searching were “adult spinal deformity” OR “adult scoliosis” OR “adult deformity” OR “degenerative scoliosis” OR “iatrogenic deformity” OR “secondary deformity.” No limitation was applied in language or article type or publishing time.

**Figure 1:**
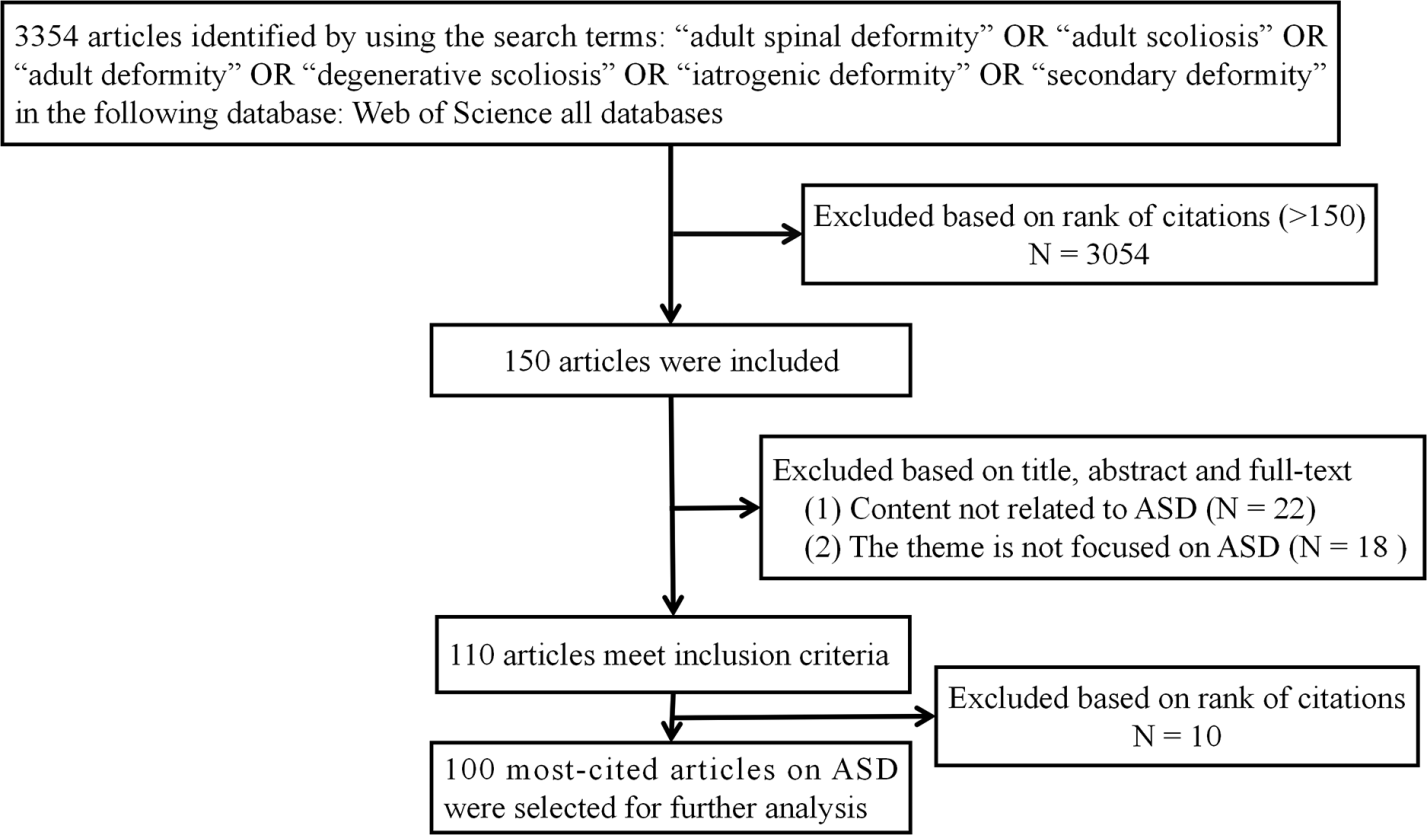
Flow diagram of literature search showing studies identified, included, and excluded at each stage.

A total of 3,354 papers matched our search criteria. Then, the results were listed from highest to lowest by “citation times.” Two reviewers independently evaluated eligible studies according to predefined inclusion and exclusion criteria and then identified the 100 most-cited articles on ASD for further analysis (Figure 1). Any dispute was resolved through consensus.

### Eligibility criteria

Inclusion criteria: (1) A clear correlation with ASD; (2) basic or clinical research associated with ASD; (3) theme focus on ASD.

Exclusion criteria: (1) The theme was not focused on ASD, such as adolescent scoliosis; (2) the types of articles are letters, news, meetings, etc.

### Data extraction

From the included 100 most-cited articles, we extracted bibliographic information such as publication title, year of publication, the institution of publication, abstract, first author, journal title, impact factor (IF), author keywords, the total number of citations, citation density, country, language, and themes. For the information extraction of the institution, we collected the first author's institution. If there were multiple affiliations of the first author, we only analyzed the ranked first.

At the same time, the same search terms were used in the identical database. Then, collect all of the literature in the recent 5 years (from 2017 to April 2022) and analyze the author keywords with VOSviewer (Version. 1.6.16) and CiteSpace (Version. 6.1R2). The previous articles can be used to learn more about how these programs work (12–15).

## Results

### Top 100 most-cited articles

The top 100 most-cited articles on ASD were selected from 3,354 papers that matched our search criteria, and all papers were written in English. The citation count among them ranged from 100 to 1,145, and the mean citation number was 215.2. Meanwhile, 32 articles were cited at least 200 times. The article “The impact of positive sagittal balance in adult spinal deformity,” published by Glassman et al., was the most-cited study (cited 1,145 times) and the highest cited density article (69.1 times per year). The papers “Adult Scoliosis and Back Pain,” published in 1979 by Nachemson et al., and “Global Alignment and Proportion (GAP) Score Development and Validation of a New Method of Analyzing Spinopelvic Alignment to Predict Mechanical Complications After Adult Spinal Deformity Surgery” published in 2017 by Yilgor et al., were the earliest and the most recent article, respectively. As shown in Figure 9A, the top 100 most-cited articles were published between 1979 and 2017, with 25 percent of the articles published before 2005. In addition, between 2005 and 2014, 73 articles were published, accounting for nearly three-quarters of the total. During this period, the largest number of articles were published in 2010, with 11 papers, followed by 10 articles in 2013. It can be inferred that this decade was very important for the development of ASD research (Table 1 and Figure 9A).

**Table 1:** The 100 most-cited articles on adult spinal deformity.

Rank	Title	First author	Year	Citations	Citations/year
1	The impact of positive sagittal balance in adult spinal deformity	Glassman, S. D.	2005	1,145	69.1
2	Correlation of radiographic parameters and clinical symptoms in adult scoliosis	Glassman, S. D.	2005	780	45.7
3	Pelvic tilt and truncal inclination two key radiographic parameters in the setting of adults with spinal deformity	Lafage, V.	2009	742	58.6
4	Adult spinal deformity-postoperative standing imbalance how much can you tolerate? An overview of key parameters in assessing alignment and planning corrective surgery	Schwab, F.	2010	653	57.6
5	Scoliosis Research Society-Schwab adult spinal deformity classification A Validation Study	Schwab, F.	2012	629	63.4
6	Radiographical spinopelvic parameters and disability in the setting of adult spinal deformity: A prospective multicenter analysis	Schwab, F.	2013	536	60.7
7	The adult scoliosis	Aebi, M.	2005	527	32.3
8	Sagittal plane considerations and the pelvis in the adult patient	Schwab, F.	2009	514	40.6
9	Adult scoliosis: Prevalence, SF-36, and nutritional parameters in an elderly volunteer population	Schwab, F.	2005	508	30.0
10	Proximal junctional kyphosis in adult spinal deformity following long instrumented posterior spinal fusion—Incidence, outcomes, and risk factor analysis	Glattes, R. C.	2005	373	22.3
11	Posterior vertebral column resection for severe spinal deformities	Suk, S.	2002	345	17.8
12	Adult spinal deformity surgery—Complications and outcomes in patients over age 60	Daubs, M. D.	2007	338	23.2
13	Adult scoliosis—A quantitative radiographic and clinical analysis	Schwab, F.	2002	326	16.2
14	Outcome and complications of long Fusions to the Sacrum in adult spine deformity—Luque-galveston, combined iliac and sacral screws, and sacral fixation	Emami, A.	2002	293	14.7
15	The impact of perioperative complications on clinical outcome in adult deformity surgery	Glassman, S. D.	2007	285	19.8
16	Instrumentation-related complications of multilevel fusions for adult spinal deformity patients over age 65—Surgical considerations and treatment option in patients with poor bone quality	DeWald, C. J.	2006	277	17.8
17	Adult scoliosis and back pain	Nachemson, A.	1979	263	6.1
18	Pseudarthrosis in long adult spinal deformity instrumentation and fusion to the sacrum: Prevalence and risk factor analysis of 144 cases	Kim, Y. J.	2006	262	16.8
19	Minimum 5-year analysis of L5-S1 fusion using sacropelvic fixation (bilateral S1 and iliac screws) for spinal deformity	Tsuchiya, K.	2006	262	16.2
20	Complications in posterior fusion and instrumentation for degenerative lumbar scoliosis	Cho, K. J.	2007	258	17.7
21	Does treatment (nonoperative and operative) improve the two-year quality of life in patients with adult symptomatic lumbar scoliosis a prospective: multicenter evidence-based medicine study	Bridwell, K. H.	2009	257	20.4
22	A prospective, nonrandomized, multicenter evaluation of extreme lateral interbody fusion for the treatment of adult degenerative scoliosis perioperative outcomes and complications	Isaacs, R. E.	2010	252	22.2
23	Proximal junctional kyphosis in adult spinal deformity after segmental posterior spinal instrumentation and fusion—Minimum five-year follow-up	Kim, Y. J.	2008	250	18.4
24	Early outcomes and safety of the minimally invasive, lateral retroperitoneal transpsoas approach for adult degenerative scoliosis	Dakwar, E.	2010	244	20.2
25	Risk–benefit assessment of surgery for adult scoliosis an analysis based on patient age	Smith, J. S.	2011	241	22.1
26	Adult degenerative scoliosis: Evaluation and management	Silva, F. E.	2010	235	19.5
27	Incidence, risk factors and classification of proximal junctional kyphosis: surgical outcomes review of adult idiopathic scoliosis	Yagi, M.	2011	234	20.8
28	The SRS–Schwab adult spinal deformity classification: assessment and clinical correlations based on a prospective operative and nonoperative cohort	Terran, J.	2013	229	26.9
29	Compensatory spinopelvic balance over the hip axis and better reliability in measuring lordosis to the pelvic radius on standing lateral radiographs of adult volunteers and patients	Jackson, R. P.	1998	223	9.4
30	Incidence, risk factors, and natural course of proximal junctional kyphosis surgical outcomes review of adult idiopathic scoliosis. Minimum 5 years of follow-up	Yagi, M.	2012	220	22.8
31	The T1 pelvic angle, a novel radiographic measure of global sagittal deformity, accounts for both spinal inclination and pelvic tilt and correlates with health-related quality of life	Protopsaltis, T.	2014	219	29.2
32	A clinical impact classification of scoliosis in the adult	Schwab, F.	2006	206	13.2
33	Defining spino-pelvic alignment thresholds should operative goals in adult spinal deformity surgery account for age?	Lafage, R.	2016	192	30.7
34	Adult scoliosis: Surgical indications, operative management, complications, and outcomes	Bradford, D. S.	1999	190	8.5
35	Predictive factors for proximal junctional kyphosis in long fusions to the sacrum in adult spinal deformity	Maruo, K.	2013	190	22.6
36	Minimally invasive multilevel percutaneous correction and fusion for adult lumbar degenerative scoliosis A Technique and Feasibility Study	Anand, N.	2008	187	13.9
37	Global Alignment and Proportion (GAP) score development and validation of a new method of analyzing spinopelvic alignment to predict mechanical complications after adult spinal deformity surgery	Yilgor, C.	2017	187	41.6
38	Impact on health related quality of life of adult spinal deformity (ASD) compared with other chronic conditions	Pellise, F.	2015	186	25.7
39	Prospective multicenter assessment of perioperative and minimum 2-year postoperative complication rates associated with adult spinal deformity surgery	Smith, J. S.	2016	186	32.4
40	Change in Classification Grade by the SRS-Schwab Adult Spinal Deformity Classification predicts impact on health-related quality of life measures prospective analysis of operative and nonoperative treatment	Smith, J. S.	2013	185	21.6
41	Improvement of back pain with operative and nonoperative treatment in adults with scoliosis	Smith, J. S.	2009	185	14.5
42	Vertebral column resection for the treatment of rigid coronal decompensation	Bradford, D. S.	1997	181	7.3
43	Complications and radiographic correction in adult scoliosis following combined transpsoas extreme lateral interbody fusion and posterior pedicle screw instrumentation	Tormenti, M. J.	2010	179	14.8
44	Complications and Risk Factors of Primary Adult Scoliosis Surgery A Multicenter Study of 306 Patients	Charosky, S.	2012	177	17.7
45	Operative vs. nonoperative treatment of leg pain in adults with scoliosis a retrospective review of a prospective multicenter database With two-year follow-up	Smith, J. S.	2009	169	13.3
46	Minimally invasive surgery for thoracolumbar spinal deformity: initial clinical experience with clinical and radiographic outcomes	Wang, M. Y.	2010	165	13.7
47	Degenerative lumbar scoliosis associated with spinal stenosis	Ploumis, A.	2007	164	11.1
48	Studies in the modified scoliosis research society outcomes instrument in adults: Validation, reliability, and discriminatory capacity	Berven, S.	2003	163	8.8
49	Long adult deformity fusions to L5 and the sacrum—A matched cohort analysis	Edwards, C. C.	2004	163	9.3
50	Patients with proximal junctional kyphosis requiring revision surgery have higher postoperative lumbar lordosis and larger sagittal balance corrections	Kim, H. J.	2014	159	19.9
51	Can c7 plumbline and gravity line predict health related quality of life in adult scoliosis?	Mac-Thiong, J.-M.	2009	158	12.4
52	Assessment of symptomatic rod fracture after posterior instrumented fusion for adult spinal deformity	Smith, J. S.	2012	157	16.5
53	Adult scoliosis surgery outcomes: A systematic review	Yadla, S.	2010	153	12.7
54	Mid-term to long-term clinical and functional outcomes of minimally invasive correction and fusion for adults with scoliosis	Anand, N.	2010	152	12.6
55	Major intraoperative neurologic deficits in pediatric and adult spinal deformity patients. Incidence and etiology at one institution	Bridwell, K. H.	1998	150	6.2
56	Proximal junctional kyphosis and failure after spinal deformity surgery	Lau, D.	2014	148	20.2
57	Adult scoliosis: A health assessment analysis by SF-36	Schwab, F.	2003	148	7.8
58	Pseudarthrosis in adult spinal deformity following multisegmental instrumentation and arthrodesis	Kim, Y. J.	2006	147	9.2
59	Risk factors for major peri-operative complications in adult spinal deformity surgery: a multi-center review of 953 consecutive patients	Schwab, F.	2012	144	15.4
60	Scoliosis research society morbidity and mortality of adult scoliosis surgery	Sansur, C. A.	2011	140	12.7
61	Incidence, mode, and location of acute proximal junctional failures after surgical treatment of adult spinal deformity	Hostin, R.	2013	139	15.6
62	Short fusion vs. long fusion for degenerative lumbar scoliosis	Cho, K. J.	2008	138	9.9
63	The SRS classification for adult spinal deformity—Building on the King/Moe and Lenke Classification Systems	Lowe, T.	2006	137	8.8
64	Prospective multicenter assessment of risk factors for rod fracture following surgery for adult spinal deformity	Smith, J. S.	2014	136	18.6
65	The validity of the SRS-22 instrument in an adult spinal deformity population compared with the Oswestry and SF-12—A study of response distribution, concurrent validity, internal consistency, and reliability	Bridwell, K. H.	2005	135	7.9
66	Pain and disability determine treatment modality for older patients with adult scoliosis, while deformity guides treatment for younger patients	Bess, S.	2009	130	10.3
67	Surgical treatment of adult scoliosis. A review of two hundred and twenty-two cases	Swank, S.	1981	128	3.1
68	Scoliosis—Surgical vs. nonsurgical treatment	Lonstein, J. E.	2006	126	7.8
69	Proximal junctional kyphosis in primary adult deformity surgery: Evaluation of 20 Degrees as a Critical Angle	Bridwell, K. H.	2013	125	14.2
70	Major Complications in Revision Adult Deformity Surgery Risk Factors and Clinical Outcomes With 2- to 7-Year Follow-up	Cho, S. K.	2012	124	12.3
71	Spino-Pelvic Parameters After Surgery Can be Predicted A Preliminary Formula and Validation of Standing Alignment	Lafage, V.	2011	124	11.5
72	Revision Rates Following Primary Adult Spinal Deformity Surgery Six Hundred Forty-Three Consecutive Patients Followed-up to Twenty-Two Years Postoperative	Pichelmann, M. A.	2010	124	10.1
73	Outcomes of Operative and Nonoperative Treatment for Adult Spinal Deformity: A Prospective, Multicenter, Propensity-Matched Cohort Assessment With Minimum 2-Year Follow-up	Smith, J. S.	2016	123	21.1
74	Changes in Radiographic and Clinical Outcomes With Primary Treatment Adult Spinal Deformity Surgeries From Two Years to Three-to Five-Years Follow-up	Bridwell, K. H.	2010	120	10.4
75	Spinal fusions to the sacrum in adults with scoliosis	Kostuik, J. P.	1983	120	3.1
76	Characterization and surgical outcomes of proximal junctional failure in surgically treated patients with adult spinal deformity	Yagi, M.	2014	120	15.2
77	Current status of adult spinal deformity	Youssef, J. A.	2013	120	13.2
78	Risk factors of sagittal decompensation after long posterior instrumentation and fusion for degenerative lumbar scoliosis	Cho, K. J.	2010	119	10.2
79	Spontaneous improvement of cervical alignment after correction of global sagittal balance following pedicle subtraction osteotomy Presented at the 2012 Joint Spine Section Meeting Clinical article	Smith, J. S.	2012	119	12.5
80	Degenerative adult onset scoliosis	Grubb, S. A.	1988	118	3.5
81	A prospective study of *de novo* scoliosis in a community based cohort	Kobayashi, T.	2006	118	7.3
82	Medical complications after adult spinal deformity surgery incidence, risk factors, and clinical impact	Soroceanu, A.	2016	117	21.6
83	Adult degenerative lumbar scoliosis	Daffner, S. D.	2003	116	6.1
84	Degenerative lumbar scoliosis: evaluation and management	Tribus, C. B.	2003	114	6.0
85	Impact of magnitude and percentage of global sagittal plane correction on health-related quality of life at 2-years follow-up	Blondel, B.	2012	113	11.7
86	Results of surgical treatment of painful adult scoliosis	Grubb, S. A.	1994	113	4.1
87	Frailty index is a significant predictor of complications and mortality after surgery for adult spinal deformity	Leven, D. M.	2016	112	21.0
88	The health impact of symptomatic adult spinal deformity: Comparison of deformity types to United States population norms and chronic diseases	Bess, S.	2016	111	18.0
89	Adult degenerative scoliosis treated with XLIF clinical and radiographical results of a prospective multicenter study with 24-month follow-up	Phillips, F. M.	2013	110	12.9
90	Adult degenerative scoliosis: A review	Birknes, J. K.	2008	109	8.0
91	A correlation of radiographic and functional measurements in adult degenerative scoliosis	Ploumis, A.	2009	107	8.4
92	Is the SRS-22 instrument responsive to change in adult scoliosis patients having primary spinal deformity surgery?	Bridwell, K. H.	2007	105	7.2
93	Scoliosis in adults aged forty years and older prevalence and relationship to age, race, and gender	Kebaish, K. M.	2011	105	9.6
94	Reoperation after primary fusion for adult spinal deformity rate, reason, and timing	Mok, J. M.	2009	105	8.1
95	Medical complications of surgical treatment of adult spinal deformity and how to avoid them	Baron, E. M.	2006	104	6.7
96	Health outcome assessment before and after adult deformity surgery. A prospective study	Albert, T. J.	1995	103	3.9
97	Osteotomies/spinal column resections in adult deformity	Enercan, M.	2013	103	11.3
98	Predicting outcome and complications in the surgical treatment of adult scoliosis	Schwab, F.	2008	102	7.5
99	Thoracolumbar deformity arthrodesis to L5 in adults: The fate of the L5-S1 disc	Edwards, C. C.	2003	101	5.4
100	Proximal junctional kyphosis results in inferior SRS pain subscores in adult deformity patients	Kim, H. J.	2013	100	11.2

### Country of origin

**Figure 2:**
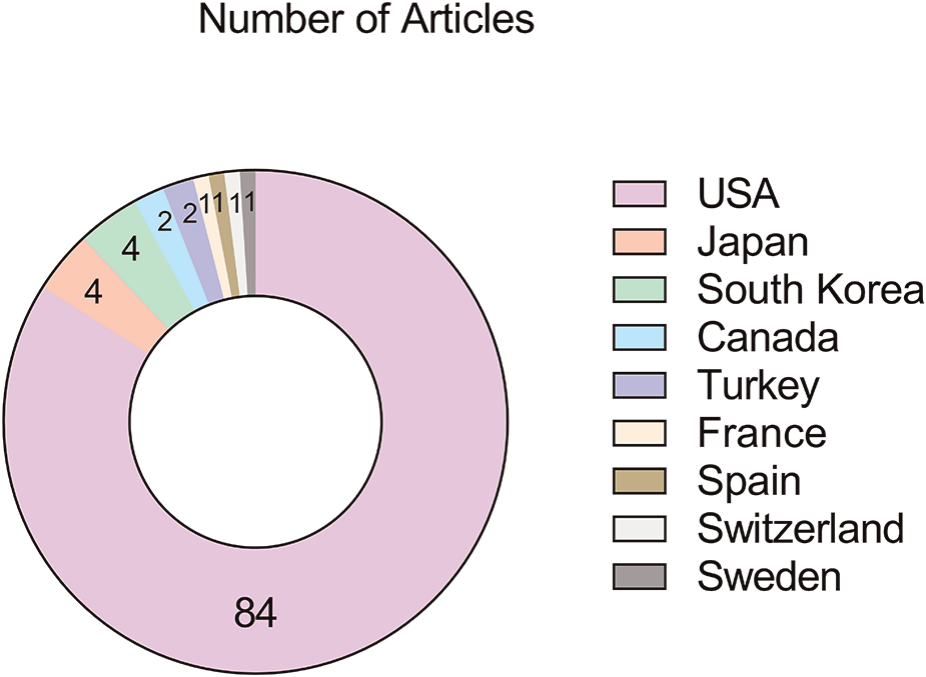
The countries of origin for the 100 most-cited articles on ASD.

The top 100 articles originated from a total of nine different countries. The United States of America had the largest number of published articles (*n* = 84) in the field of ASD; followed by Japan and South Korea tied for second place (*n* = 4, each); and then, Canada and Turkey each contributed two papers; countries from France, Spain, Sweden, and Switzerland published one article each. Judging by the results, most of them came from developed countries in Europe and the United States (Figure 2).

### The highly productive first author and relationship between authors

A total of 63 different first authors contributed to the top 100 articles, and 15 authors published 2 or more articles. The top 3foremost productive first authors were Schwab F., Smith J. S., and Bridwell K. H., with published 10, 9, and 6 articles respectively. It is worth mentioning that, although Glassman S. D. only contributed to three articles, he had the highest mean citation number, reaching 736.7 per article (Table 2 and Figure 8).

**Table 2:** First authors with two or more papers of the 100 most-cited articles on adult spinal deformity.

Authors (first)	Number of article	Number of total citations	Number of mean citation	Country
Schwab F.	10	3,766	376.6	United States
Smith J. S.	9	1,501	166.8	United States
Bridwell K. H.	6	892	148.7	United States
Glassman S. D.	3	2,210	736.7	United States
Kim Y. J.	3	659	219.7	United States
Yagi M.	3	574	191.3	Japan
Cho K. J.	3	515	171.7	South Korea
Lafage V.	2	866	433	United States
Bradford D. S.	2	371	185.5	United States
Anand N.	2	339	169.5	United States
Ploumis A.	2	271	135.5	United States
Edwards C. C.	2	264	132	United States
Kin H. J.	2	259	129.5	United States
Bess S.	2	241	120.5	United StatesUnited States
Grubb S. A.	2	231	115.5	United States

We used Bibliographic coupling, Citation, and Co-authorship analysis to explore the relationships among the authors of the top 100 articles. As shown in Figure 8, a total of 365 authors were recorded, with 22 authors appearing in more than 5 documents at the same time chosen and analyzed. In Figure 8A, the Bibliographic coupling analysis of authors showed that the top five authors with the total link strength were as follows: Schwab, Frank J. (total link strength = 22,894), Shaffrey, Christopher I. (total link strength = 19,701), Lafage, Virginie (total link strength = 16,577), Smith, Justin S. (total link strength = 15,677) and Bess, Shay (total link strength = 14,363). The figure also showed that Schwab, frank j had a strong connection to Lafage, Virginie, Smith, Justin S., Shaffrey, Christopher I., Bess, Shay, Hostin, Richard, Hart, Robert, and Klineberg, Eric. In Figure 8B, the Citation analysis of authors showed that the top five authors with the total link strength were as follows: Schwab, Frank J. (total link strength = 1,646), Shaffrey, Christopher I (total link strength = 1,284), Lafage, Virginie (total link strength = 1,010), Smith, Justin S. (total link strength = 953), and Berven, Sigurd H. (total link strength = 831). The figure also showed that Schwab, Frank J. had a strong connection to Lafage, Virginie, Hostin, Richard, Bess, Shay, Smith, Justin S., and Shaffrey, Christopher I. Similarly, in Figure 8C, the co-authorship analysis of authors showed that the top five authors with the total link strength were as follows: Schwab, Frank J. (total link strength = 170), Shaffrey, Christopher I. (total link strength = 156), Smith, Justin S. (total link strength = 125), Bess, Shay (total link strength = 124), and Lafage, Virginie (total link strength = 121). The figure also showed that Schwab, Frank J. had a strong connection to Smith, Justin S., Lafage, Virginie, Bess, Shay, Hostin, Richard, and Shaffrey, Christopher I. The above results suggested that the stronger the connection between the authors, the closer the cooperation between them.

### Institutions of publication

In total, 41 different institutions contributed to the top 100 articles, with 15 institutions publishing two or more. The top three institutions were the University of Washington (*n* = 15), New York University (*n* = 13), and University of Virginia (*n* = 9), with 2,912, 4,308, and 1,501 total citations, respectively. Among the 15 institutions which published two or more articles, the average number of citations exceeded 100 times, with the University of Louisville having the most, reaching 736.7 times (Table 3).

**Table 3:** The institutions providing two papers or more to the most highly cited ASD articles.

Institution (first)	Articles (*n*)	Number of total citations	Number of mean citation	Country
University of Washington	15	2,912	194.1	United States
New York University	13	4,308	331.4	United States
University of Virginia	9	1,501	166.8	United States
University of California San Francisco	7	1,270	181.4	United States
Thomas Jefferson University	5	585	117	United States
Inje University	4	860	215	South Korea
University of Louisville	3	2,210	736.7	United States
Maimonides Hospital	3	982	327.3	United States
Twin Cities Spine Center	3	429	143	United States
Rush University	2	387	193.5	United States
National Hospital Organization Murayama Medical Center	2	340	170	Japan
Cedars Sinai Medical Center	2	339	169.5	United States
Hospital for Special Surgery	2	259	129.5	United States
University of Minnesota	2	254	127	United States
North Carolina Spine Center	2	231	115.5	United States

### Journals of publication

The top 100 most-cited articles were collected in 13 different journals, with at least two studies published in six of them. *Spine* was the most contributed journal, publishing more than two-thirds (*n* = 69) of the articles, with a total of 16,308 citations. *Neurosurgery* (*n* = 7) was the second most popular journal, followed by *Neurosurgical Focus* (*n* = 6) and *European Spine Journal* (*n* = 5), with citation counts of 1,041, 1,128, and 1,098, respectively (Table 4).

**Table 4:** The journals in which the top-cited 100 articles were published.

Journal	Articles (*n*)	Number of total citations	Number of mean citation	I.F.
*Spine*	69	16,308	236.3	2.646
*Neurosurgery*	7	1,041	148.7	4.853
*Neurosurgical Focus*	6	1,128	188.0	3.642
*European Spine Journal*	5	1,098	219.6	2.458
*Journal of Bone and Joint Surgery-American Volume*	3	553	184.3	4.578
*Journal of Neurosurgery-Spine*	3	441	147.0	3.011
*Journal of Spinal Disorders & Techniques*	1	187	187.0	2.31
*Spine Journal*	1	164	164.0	3.191
*The Journal of Bone and Joint Surgery. American volume*	1	128	128.0	NA
*Clinical Orthopaedics and Related Research*	1	126	126.0	4.329
*Global Spine Journal*	1	120	120.0	2.683
*American Journal of Orthopedics (Belle Mead, N.J.)*	1	116	116.0	NA
*The Journal of the American Academy of Orthopaedic Surgeons*	1	114	114.0	2.286

### Themes distribution

**Figure 3:**
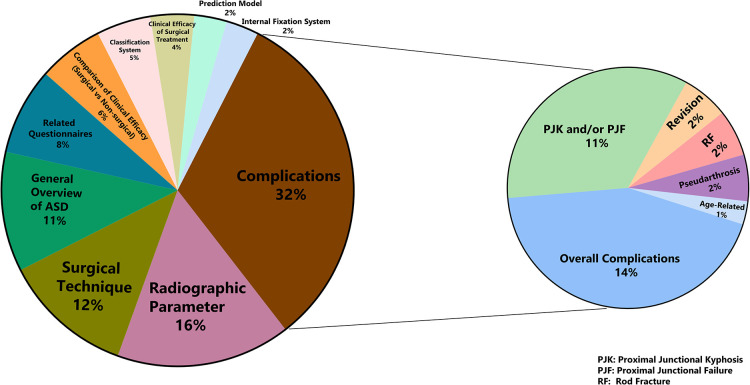
The themes for the 100 most-cited articles on ASD.

The top 100 articles were composed of 83 original articles, 15 reviews or systematic reviews, and two case series. The majority of study designs were retrospective and prospective cohort studies, with many coming from multicenter databases. Taken together, there were 10 different core themes around the top 100 articles. Complications (*n* = 32) were the most studied themes, followed by radiographic parameters (*n* = 16), surgical techniques (*n* = 12), general overview of ASD (*n* = 11), related questionnaires (*n* = 8), comparison of clinical efficacy (surgical vs. non-surgical) (*n* = 6), classification system (*n* = 5) and clinical efficacy of surgical treatment (*n* = 4). As for the prediction model (*n* = 2) and internal fixation system (*n* = 2), it was less involved. In the theme of complications, proximal junctional kyphosis (PJK) and proximal junctional failure (PJF) were the main research content, except for the overall complications (Figure 3).

### Author keywords analysis and visualization

**Figure 4:**
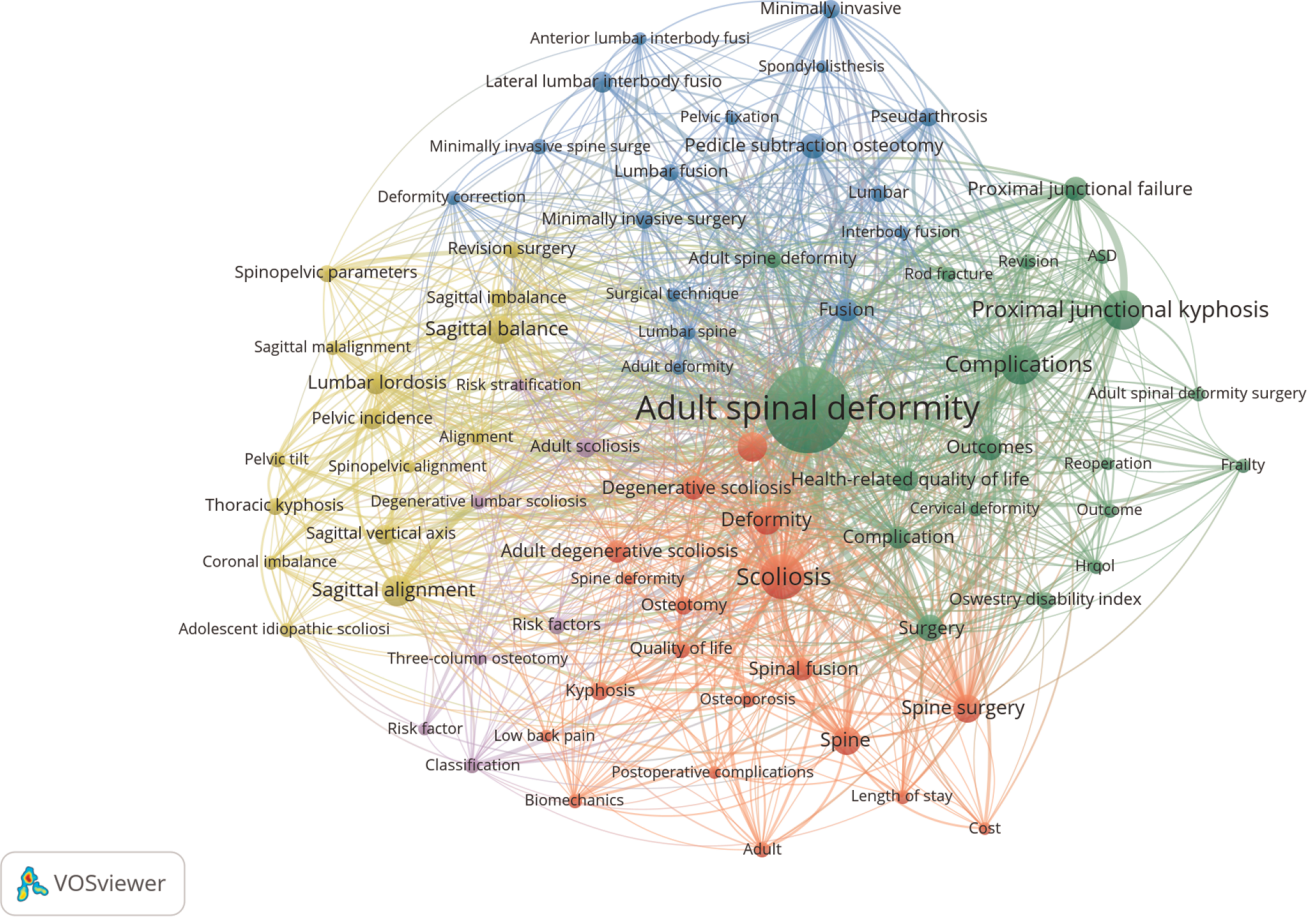
The network visualization analysis of author keywords of the whole articles published in the recent 5 years (overlay visualization).

**Figure 5:**
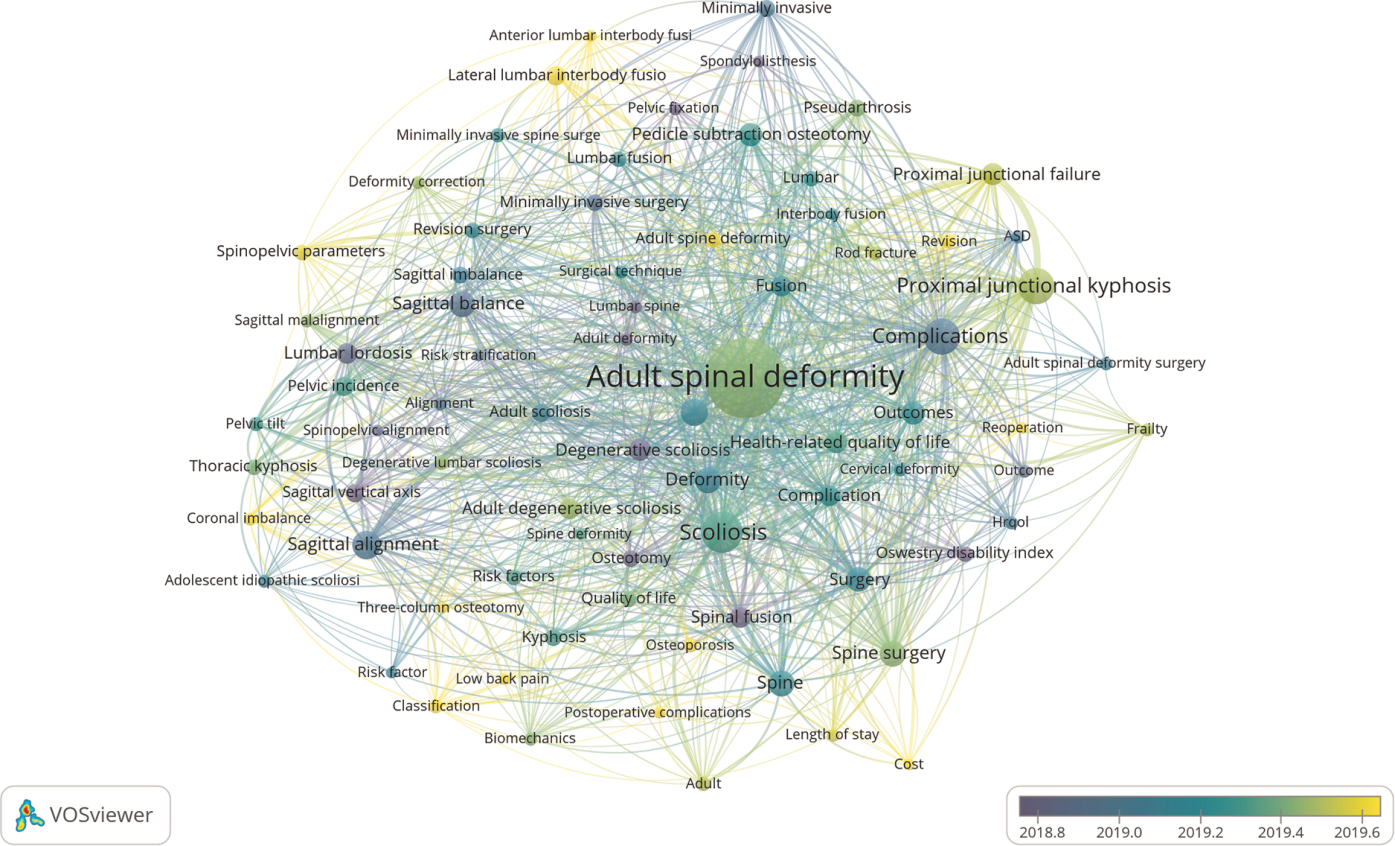
The network visualization analysis of author keywords of the whole articles published in the recent 5 years (time-dependent overlay visualization).

In the recent 5 years, a total of 1,993 articles have been published. According to the Co-Occurrence Network and Cluster Analysis based on VOSviewer, there were 3,060 author keywords extracted from the above articles and 78 keywords repeated at least 15 times. The author keywords co-occurrence visual network diagram showed five different clusters roughly in Figure 4. Distinctive color represented different clusters, and the line between each keyword implied co-occurrence in the same articles. The cluster on yellow mainly focused on the sagittal plane parameter. “Sagittal alignment” occurrences times was 94 and “sagittal balance” was 74; cluster on blue mainly concentrated in surgical technique, “pedicle subtraction osteotomy” occurrences times was 47, and “minimally invasive” and “minimally invasive surgery” was 34 and 33, respectively; cluster on green focused on complications, “complications” and “complication” occurrences times were 146 and 47, respectively. In addition, “PJK” occurrences times was 145, “PJF” was 57 and pseudarthrosis was 34 (Supplementary Table S1). While cluster of red and purple focused on risk factors and cost. Figure 5 showed author keywords from 2017 to 2022, in which the color implied the time when the keywords appeared, and the yellower the color in the picture, the newer the keywords. The picture showed that “reoperation,” “PJK,” “revision,” “rod fracture,” “PJF,” “Lateral lumbar interbody fusion,” “spinopelvic parameters”, and “coronal imbalance” were the latest research hotspots.

**Figure 6:**
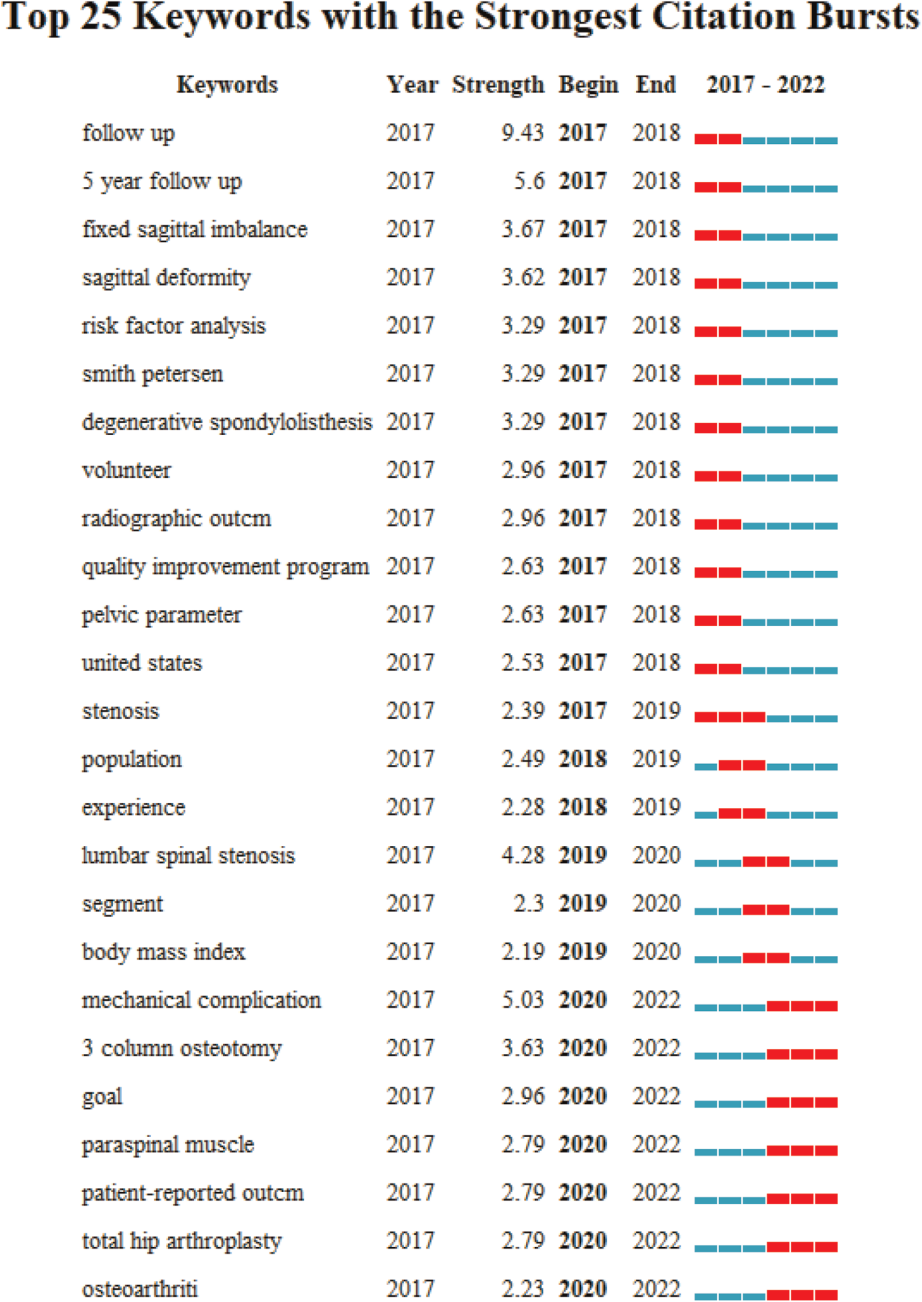
Top 25 author keywords with strongest citation bursts based on CiteSpace (from 2017 to 2022).

**Figure 7:**
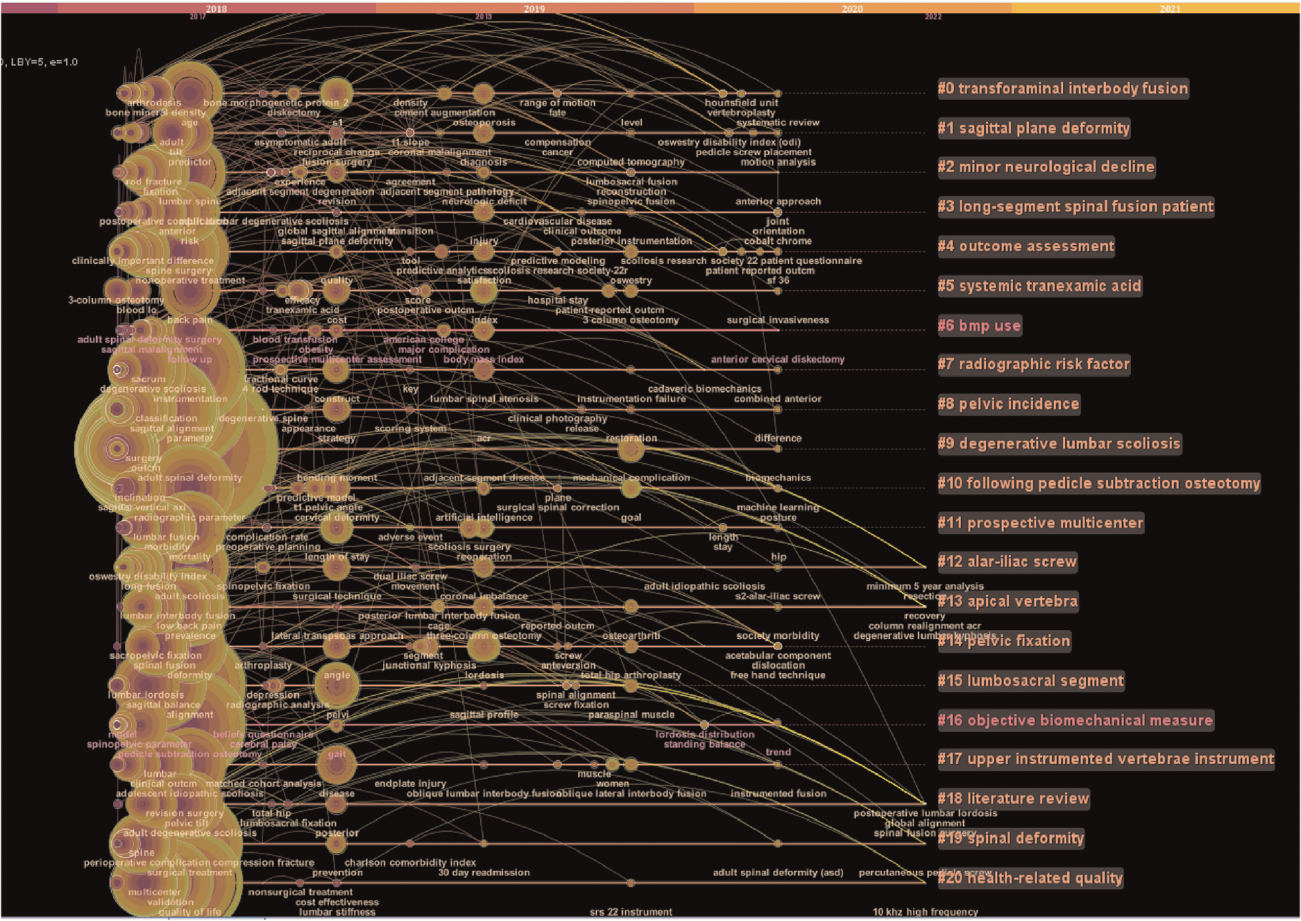
Timeline visualization of author keywords based on CiteSpace (from 2017 to 2022). The node represents the time when the keyword first occurs. The larger the node, the more times it appears. The clustering label was obtained by CiteSpace software according to the timeline on the right of the figure. Each row in the figure shows the occurrence of the keyword in different years. The line between each node represented the correlation and the different colors implied the diverse time, the purple line was the oldest (2017), while the yellow line was the newest (2022).

Additionally, author keywords burst and timeline visualization were displayed by CiteSpace (time-space from January 2017 to April 2022), which were usually employed to identify research trends and hot topics. Figure 6 showed the top 25 author keywords with the strongest citation bursts. The red bar represents the strongest citation bursts. The keywords burst from 2020 to 2022 were “mechanical complication,” “3 column osteotomy,” “goal,” “paraspinal muscle,” “patient-reported outcome,” and so on. These keywords indicated the hotspots of research in the recent two years. In addition, the keywords timeline view demonstrated the evolution of the research trend (Figure 7). The larger the nodes, the more published articles, and the most recent studies were nearly closer to the right. The line between each node represented the correlation and the different colors implied the diverse time, with purple line being the oldest (2017), and the yellow line being the newest (2022). In this picture, we could find that the keywords such as “machine learning,” “S2-alar-iliac-screw,” “muscle,” and “paraspinal muscle” were close to the right of the picture and appear relatively recently, suggesting that these topics had received more attention recently.

## Discussion

In the present study, we used bibliometric methods to analyze the most influential articles on ASD from the Web of Science database, which is a trustworthy database that allows for making citation analysis. We found that the top 100 most-cited articles on ASD were published primarily in the journal of *spine* and they were mainly from Western developed countries, particularly in the United States. The most productive first author was Schwab Frank, who came from New York University. In the past, the most important topics in the field of ASD were complications, sagittal plane parameters, and surgical techniques had been. The papers reported similar themes from the literature in the last 5 years when taking the author keywords into account. Complications and sagittal balance remain the focus of current research. However, since 2020, “3 column osteotomy” and “paraspinal muscle” displayed citation bursts, implying that there have been great changes in this field in a short time. In a nutshell, these messages provided a comprehensive understanding of the most important studies, discussed and topical in the field of ASD. Moreover, this study may help guide classic journal selection, hotspot tracking, and research trend catching for some scholars who are interested in ASD.

Among the top 100 most-cited articles on ASD, publication dates ranged from 1979 to 2017. Only 10 of the most influential articles were published before the year 2000. Subsequently, the number of articles was continuously increasing, between 2001 and 2010 up to 54. While 36 studies were finished between 2011 and 2017. The oldest article of the top 100 most-cited studies on ASD was recorded in the journal of *spine* in 1979, written by Nachemson A., describing the relationship between adult scoliosis and back pain (8). The newest article in our study was published in the *Journal of Bone and Joint Surgery-American Volume* by Yilgor C. in 2017 (16). In this study, the authors proposed a new pelvic-incidence-based proportional method, global alignment and proportion (GAP) score, which could effectively predict mechanical complications in ASD. The most-cited article was written by Glassman S. D., who came from the University of Louisville (17). The authors performed a prospective multicentric study with the correlation between positive sagittal balance and health status. They found that positive sagittal balance was linearly correlated with clinical symptoms. The reason for this most frequently cited article may be attributed to the subsequent understanding of sagittal balance. The scholars found that restoring the sagittal balance was more significant than correcting the coronal balance when taking HRQOL into account (18, 19). Meanwhile, we should treat the citations of articles rationally. Apart from self-citation, many factors affect the citation rate of an article, such as hotspot research, famous journals, authors with high prestige and respect for the pioneers, etc. (20, 21). In the scientific literature citations, scholars tend to cite the articles which have a high influence on the field they are studying, but also are inclined to cite the study completed by highly reputed authors who are active in this field. Therefore, there is a tendency that highly-cited articles to become even more cited, which is the so-called Matthew effect (the rich become richer) (21, 22). The Matthew effect was widely found in literature citations and affected the authenticity of citations (20, 23–25). In the scientific literature, highly cited articles may persist due to the “Matthew effect,” in which well-researched articles maintain prominence. As a result, this phenomenon will detriment to our understanding of important articles which are not well known, and will also have a negative impact on the potential for innovation and exploration in research. In addition, there is very meaningful to analyze trends in the growth of scientific literature. Generally speaking, the trend often shows slow growth at first, then exponential increase, and finally gradually reaches a stable level of growth. As we know, there are many kinds of growth models proposed in scientific literature. Common models include the exponential growth model (26, 27), logistic curve model (28, 29), and linear model (30, 31). In this study, we developed an exponential growth model and logistic regression curve to predict the number of ASD publications in the future. As shown in Figures 9B,C, the model of the exponential curve fitted the number of annual publications well (Figure 9B, *R*^2^ = 0.978). In the predictive curve (Figure 9C), it can be seen that the number of publications with ASD will reach 9,108 in 2,040, and in 2,050 the number will reach 43,055. Meanwhile, in the logistic regression curve, we could also see that the curve fits the current data very well (Figure 9D, *R*^2 ^= 0.992). However, the predictive curve showed that the number of publications will reach its peak in 2030, with about 524 papers. Since then, the annual publications have remained at this level (Figure 9E). This prediction result was quite different from the exponential model, which may suggest that larger data were needed to modify these models. In addition, the logistic model may indicate that ASD-related research will be in a bottleneck period in 2030, so it is particularly important to explore new theories to re-lead the research hotspots in this field.

**Figure 8:**
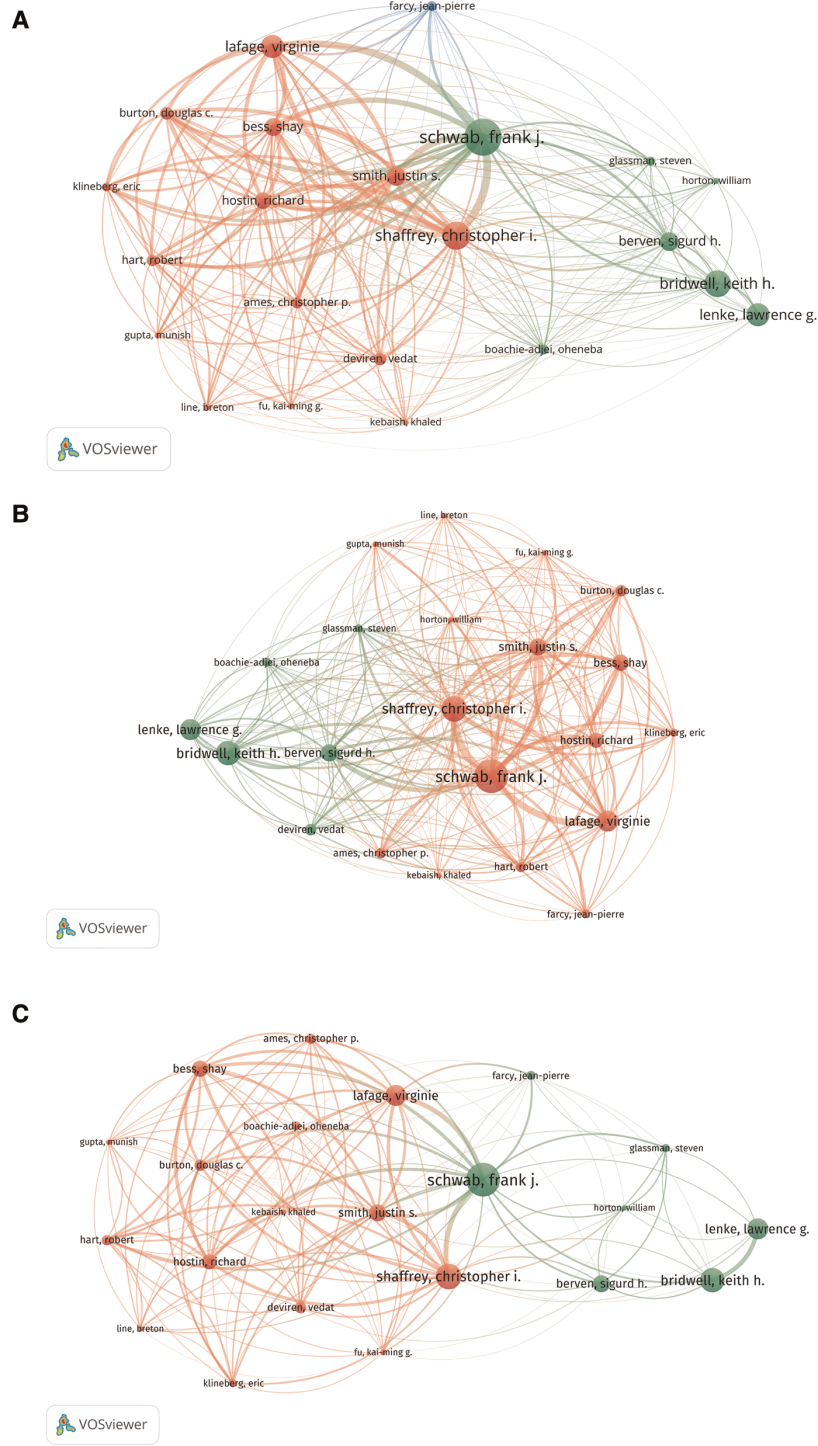
Visual mapping of the authors’ relationships. Bibliographic coupling analysis of authors from the top 100 most-cited articles on ASD (**A**); citation analysis of authors from the top 100 most-cited articles on ASD (**B**); co-authorship analysis of authors from the top 100 most-cited articles on ASD (**C**).

**Figure 9:**
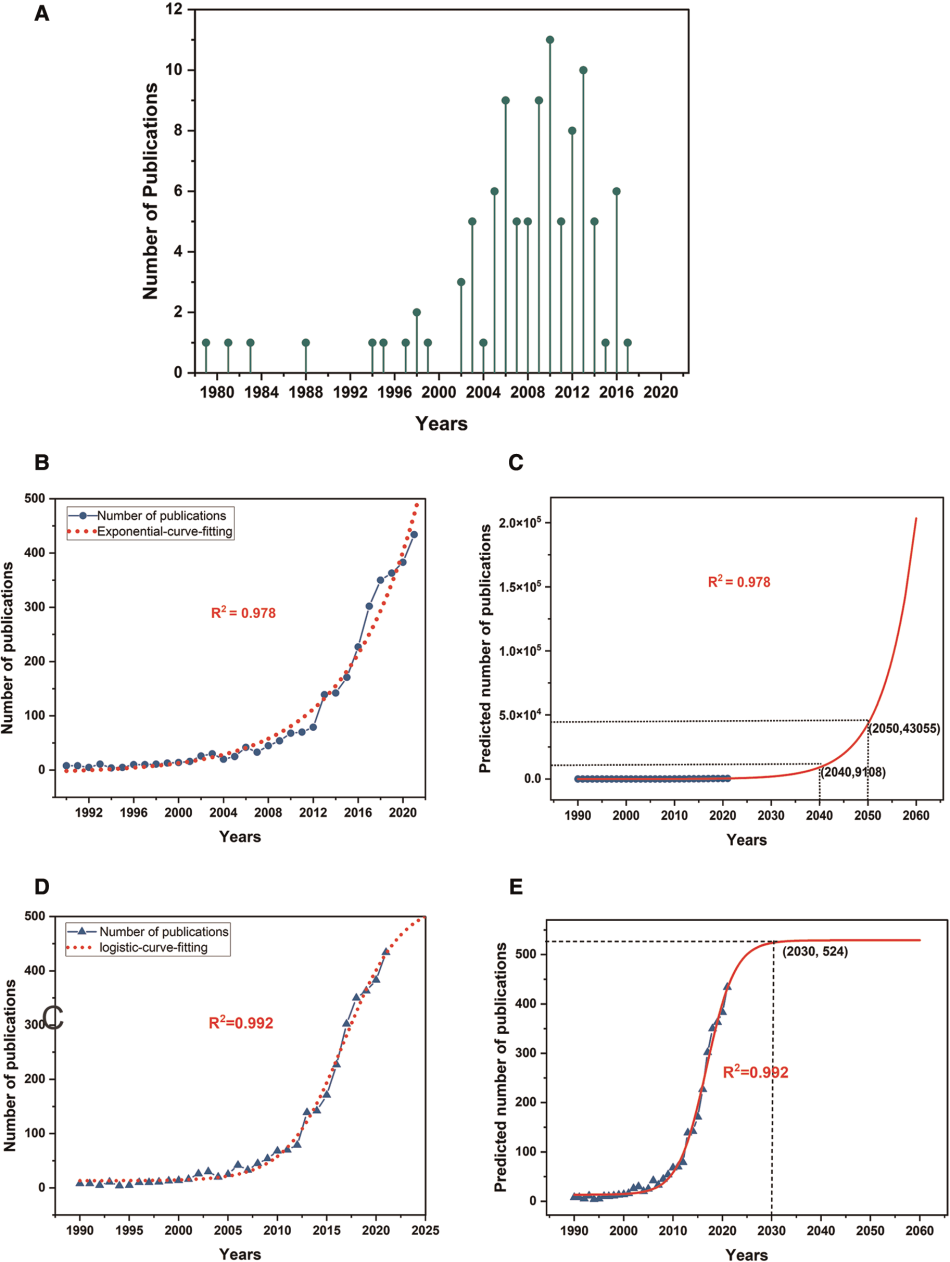
The distribution of the top 100 most-cited articles on ASD (**A**). Model of exponential fitting curves of the trends in publications related to ASD research per year (**B,C**). Model of logistic regression fitting curves of the trends in publications related to ASD research per year (**D,E**).

The 100 most-cited articles on ASD were recorded in 13 different journals. The top three journals account for more than four-fifths of the total. *Spine* was the most contributed journal. Sixty-two different first authors participated in these studies, and they came from 41 different affiliated institutions, covering nine diverse countries. The percentage of published papers in the top five first author affiliations was 49%, and the institutions were all located in the United States. Among the first author institutions, the University of Washington was the most prolific college. From this, we recognize that Western countries, especially the United States, have a certain degree of leadership in the field of ASD research. Some authors or institutions can be good candidates for academic collaboration, such as Schwab F., Smith J. S., Bridwell K. H., and Glassman S. D.

Surgical complications were the most frequently discussed themes in the top 100 most-cited articles on ASD. In our study, there were 32 articles published about complications. Fourteen of them were reported overall complications, followed by PJK and/or PJF (*n* = 11), revision (*n* = 2), rod fracture (RF) (*n* = 2), pseudarthrosis (*n* = 2), and age-related (*n* = 1). Previous multicentric studies had demonstrated that the overall complication rates ranged from 13.4% to 39%, and approximately 26% of these patients required revision surgery because of mechanical or neurological complications (7, 32). As a specific complication, PJK has a high incidence and the most likely reason for it may be that corrective surgery reconstructed the deformed spinal alignment and adjusted the direction of the stress and muscle strength which dramatically increased the stress of the junctional segments (33). In recent years, some articles had discussed the occurrence of postoperative PJK in the form of meta-analysis. Zhao et al. (34), included 55 papers for meta-analysis and found that the incidence of PJK ranged from 13.16% to 61.7% in different studies and the elderly female who accompanied osteoporosis, high sagittal vertical axis, large pelvic tilt, low lumbar lordosis, and fixed to pelvis were more suffer from PJK. Meanwhile, excessive correction of the sagittal vertical axis and lumbar lordosis were also risk factors for PJK. Zou et al. (35) explored the characteristics and risk factors of PJK; they also found that elderly female ASD patients with low bone mineral density were more susceptible to PJK and the ASD patients had larger parameters (such as proximal junctional angle, thoracic kyphosis, and sagittal alignment) except the pelvic incidence minus lumbar lordosis. To avoid the appearance of PJK, they had some suggestions as follows: upper instrumented vertebra should be fixed above T8 and used hooks instead of pedicle screws; try to avoid fixation to the pelvis; a good sagittal alignment should be obtained after correction surgery. PJK would seriously affect the HRQOL of patients, so it needs to be an adequate evaluation before the operation to minimize its incidence.

The second most discussed themes were radiographic parameters, accounting for 16% of the 100 most-cited articles. Among the radiographic parameters, many of them were sagittal plane parameters, and the correlation between sagittal parameters and clinical symptoms or outcomes was discussed in these studies. The two most representative papers were written by Glassman S. D. (17, 36), which revealed that sagittal balance was more important than coronal plane balance because the sagittal balance was more correlated with the patient's clinical symptoms. In recent years, however, the coronal imbalance had also attracted much attention. Many scholars had explored the correction techniques (37–41), risk factors (42–44), radiological parameters (19, 45, 46), and classification systems (47, 48) of coronal imbalance in ASD. Makhni et al. (40) first proposed a “kickstand rod” technique to correct coronal deformity which achieved good radiological parameters and clinical outcomes.

As for surgical techniques, fusion segment and osteotomy were mainly explored before 2008 (49–51), and since then, with the deepening of the concept of minimally invasive, a variety of minimally invasive techniques had been created, such as MIS XLIF (52) and MIS TLIF (53). Minimally invasive surgery had the characteristics of fewer complications and rapid recovery, which was in line with the patient's pursuit of rapid recovery and would be the direction of future efforts in this field.

Keywords in the recent literature can well reveal the current research hotspots and trends. We analyzed the author keywords of 1,993 articles on ASD published after 2017 and understood the current research status to some extent. Apart from the keywords describing the definition of ASD (such as “Adult spinal deformity”, “Scoliosis” and “Spinal deformity”), other keywords such as “Complications (occurrence times, *n* = 146),” “Proximal junctional kyphosis (*n* = 145),” “Sagittal alignment (*n* = 94),” “Spinal surgery (*n* = 78),” “Sagittal balance (*n* = 74),” “Surgery (*n* = 69),” “Outcomes (*n* = 63),” “Pedicle subtraction osteotomy (*n* = 61),” “Proximal junctional failure (*n* = 57),” “Health-related quality of life (*n* = 54),” “Spinal fusion (*n* = 49),” “Fusion (*n* = 48),” “Complication (*n* = 47),” “Lumbar lordosis (*n* = 47),” and “Pelvic incidence (*n* = 45)” were the 15 most popular words (Supplementary Table S1). From these most prevalent keywords, it is not difficult to find that complication, sagittal plane parameters, and surgical techniques are still the focus of current research when compared with the top 100 most-cited articles on ASD. The visualization analysis of keywords burst showed that the keywords such as “mechanical complication,” “3 column osteotomy,” “paraspinal muscle,” and “total hip arthroplasty” appeared to burst, which implied that they may be the research hotspots and emerging trends since 2020. Meanwhile, from the timeline view of keywords, we could also find that the keywords such as “paraspinal muscle,” “muscle,” “freehand technique,” and “machine learning” were much closer to the right of Figure 7, which means that there were more recent studies in these fields. Hyun et al. (54) found that patients with PJK had lower muscularity and higher fatty degeneration in long segment fusion of ASD compared to the patients without PJK. Pennington et al. (55) found that paraspinal muscle size was an independent risk factor for PJK in ASD. In addition, Bae et al. (56) found that the quality of paraspinal muscle was related to patient maintenance of upright posture and sagittal decompensation in ASD. As an important auxiliary structure to maintain the biomechanical balance of the spine (57), the paraspinal muscle should be paid more attention to in the deformity correction of ASD.

Comprehensive analysis of keywords in the collected articles could provide useful information for revealing the theme of the literature and determining the research hotspots and trends. In this study, we made a comparative analysis of the themes of the top 100 most-cited papers on ASD and the author keywords in the articles published in the last 5 years. We have found some research hotspots and trends in the field of ASD, which were summarized as follows: (1) complications and sagittal plane parameters are still the major topics of study at present and even later; (2) the study of coronal balance and paraspinal muscle is expected to be strengthened in the coming years; (3) less trauma, quick recovery, and reliable curative effects are the common goals pursued by doctors and patients. Minimally invasive surgery will continue to develop rapidly.

Inevitably, there were several limitations in this study. First, this study was only based on the Web of Science database, and some influential articles recorded in other sources may be omitted. Second, the total citation counts of the articles did not fully reflect the quality of the paper. Finally, this study is not fair to the newly published high-quality papers, and it is obvious that limited by the total number of citations, the inclusion criteria will be more conducive to earlier research.

## Conclusion

Based on a comparative analysis of the results of bibliometric and visualization, complications and sagittal plane parameters are still the major topics of research at present and even later, and minimally invasive surgery has a growth trend in this field of ASD.

## Data Availability

The datasets presented in this study can be found in online repositories. The names of the repository/repositories and accession number(s) can be found in the article/**Supplementary Material**.
