# Supplementary material for: The top 100 most-cited articles on adult spinal deformity: The most popular topics are still sagittal plane parameters and complications
Source: Front Surg. 2023 Jan 6;9:961582. doi: 10.3389/fsurg.2022.961582 (PMC9852520; doi:10.3389/fsurg.2022.961582)
Supplement: Supplementary file 1 [file Table1.doc]

**Supplementary information**

**Table 1** Top 30 Keywords in all articles Ranked by Occurrences

| **Keywords** | **Links** | **Total link strength** | **Occurrences** | **Average publication year** | **Average citations** |
| --- | --- | --- | --- | --- | --- |
| Adult spinal deformity | 74 | 1242 | 690 | 2019.41 | 6.9623 |
| Scoliosis | 67 | 450 | 189 | 2019.32 | 6.2804 |
| Complications | 63 | 369 | 146 | 2018.97 | 10.7329 |
| Proximal junctional kyphosis | 58 | 325 | 145 | 2019.46 | 9.0138 |
| Sagittal alignment | 52 | 204 | 94 | 2019 | 9.5426 |
| Spinal deformity | 55 | 163 | 81 | 2019.13 | 6.7531 |
| Spine surgery | 49 | 199 | 78 | 2019.41 | 8.9744 |
| Deformity | 59 | 196 | 78 | 2019.1 | 6.4872 |
| Sagittal balance | 51 | 178 | 74 | 2018.94 | 7.2838 |
| Spine | 46 | 154 | 74 | 2019.19 | 5.0541 |
| Surgery | 45 | 185 | 69 | 2019.14 | 7.1739 |
| Outcomes | 54 | 207 | 63 | 2019.21 | 7.9683 |
| Pedicle subtraction osteotomy | 46 | 153 | 61 | 2019.27 | 7.4426 |
| Proximal junctional failure | 35 | 154 | 57 | 2019.48 | 7.9474 |
| Degenerative scoliosis | 38 | 78 | 56 | 2018.83 | 5.4107 |
| Health-related quality of life | 49 | 149 | 54 | 2019.31 | 7.0926 |
| Spinal fusion | 39 | 103 | 49 | 2018.51 | 9.4898 |
| Fusion | 55 | 154 | 48 | 2019.18 | 11.3333 |
| Adult degenerative scoliosis | 34 | 48 | 48 | 2019.44 | 5.25 |
| Complication | 47 | 139 | 47 | 2019.22 | 10.383 |
| Lumbar lordosis | 40 | 124 | 47 | 2018.87 | 7.6809 |
| Pelvic incidence | 32 | 100 | 45 | 2019.3 | 6.9556 |
| Lateral lumbar interbody fusion | 29 | 75 | 42 | 2019.58 | 5.9048 |
| Adult scoliosis | 38 | 70 | 37 | 2019.08 | 7.5676 |
| Sagittal vertical axis | 31 | 92 | 36 | 2018.66 | 6.1111 |
| Pseudarthrosis | 40 | 108 | 34 | 2019.41 | 8.7647 |
| Kyphosis | 35 | 89 | 34 | 2019.29 | 4.5294 |
| Osteotomy | 37 | 89 | 34 | 2018.82 | 9.9706 |
| Minimally invasive | 30 | 81 | 34 | 2019 | 8.8824 |
| Minimally invasive surgery | 34 | 70 | 33 | 2018.94 | 5.6667 |

**Supplemental references(1-100).** References list of the top 100 most-cited articles on ASD.

1. Glassman SD, Bridwell K, Dimar JR, Horton W, Berven S, Schwab F. The impact of positive sagittal balance in adult spinal deformity. Spine. 2005;30(18):2024-9. doi: 10.1097/01.brs.0000179086.30449.96

2. Glassman SD, Berven S, Bridwell K, Horton W, Dimar JR. Correlation of radiographic parameters and clinical symptoms in adult scoliosis. Spine. 2005;30(6):682-8. doi: 10.1097/01.brs.0000155425.04536.f7

3. Lafage V, Schwab F, Patel A, Hawkinson N, Farcy JP. Pelvic Tilt and Truncal Inclination Two Key Radiographic Parameters in the Setting of Adults With Spinal Deformity. Spine. 2009;34(17):E599-E606. doi: 10.1097/BRS.0b013e3181aad219

4. Schwab F, Patel A, Ungar B, Farcy JP, Lafage V. Adult Spinal Deformity-Postoperative Standing Imbalance How Much Can You Tolerate? An Overview of Key Parameters in Assessing Alignment and Planning Corrective Surgery. Spine. 2010;35(25):2224-31. doi: 10.1097/BRS.0b013e3181ee6bd4

5. Schwab F, Ungar B, Blondel B, Buchowski J, Coe J, Deinlein D, et al. Scoliosis Research Society-Schwab Adult Spinal Deformity Classification A Validation Study. Spine. 2012;37(12):1077-82. doi: 10.1097/BRS.0b013e31823e15e2

6. Schwab FJ, Blondel B, Bess S, Hostin R, Shaffrey CI, Smith JS, et al. Radiographical Spinopelvic Parameters and Disability in the Setting of Adult Spinal Deformity A Prospective Multicenter Analysis. Spine. 2013;38(13):E803-E12. doi: 10.1097/BRS.0b013e318292b7b9

7. Aebi M. The adult scoliosis. European Spine Journal. 2005;14(10):925-48. doi: 10.1007/s00586-005-1053-9

8. Schwab F, Lafage V, Patel A, Farcy JP. Sagittal Plane Considerations and the Pelvis in the Adult Patient. Spine. 2009;34(17):1828-33. doi: 10.1097/BRS.0b013e3181a13c08

9. Schwab F, Dubey A, Gamez L, El Fegoun AB, Hwang K, Pagala M, et al. Adult scoliosis: Prevalence, SF-36, and nutritional parameters in an elderly volunteer population. Spine. 2005;30(9):1082-5. doi: 10.1097/01.brs.0000160842.43482.cd

10. Glattes RC, Bridwell KH, Lenke LG, Kim YJ, Rinella A, Edwards C. Proximal junctional kyphosis in adult spinal deformity following long instrumented posterior spinal fusion - Incidence, outcomes, and risk factor analysis. Spine. 2005;30(14):1643-9. doi: 10.1097/01.brs.0000169451.76359.49

11. Suk S, Kim JH, Kim WJ, Lee SM, Chung ER, Nah KH. Posterior vertebral column resection for severe spinal deformities. Spine. 2002;27(21):2374-82. doi: 10.1097/00007632-200211010-00012

12. Daubs MD, Lenke LG, Cheh G, Stobbs G, Bridwell KH. Adult spinal deformity surgery - Complications and outcomes in patients over age 60. Spine. 2007;32(20):2238-44. doi: 10.1097/BRS.0b013e31814cf24a

13. Schwab FJ, Smith VA, Biserni M, Gamez L, Farcy JPC, Pagala M. Adult scoliosis - A quantitative radiographic and clinical analysis. Spine. 2002;27(4):387-92. doi: 10.1097/00007632-200202150-00012

14. Emami A, Deviren V, Berven S, Smith JA, Hu SS, Bradford DS. Outcome and complications of long Fusions to the Sacrum in adult spine deformity - Luque-galveston, combined iliac and sacral screws, and sacral fixation. Spine. 2002;27(7):776-86. doi: 10.1097/00007632-200204010-00017

15. Glassman SD, Hamill CL, Bridwell KH, Schwab FJ, Dimar JR, Lowe TG. The impact of perioperative complications on clinical outcome in adult deformity surgery. Spine. 2007;32(24):2764-70. doi: 10.1097/BRS.0b013e31815a7644

16. DeWald CJ, Stanley T. Instrumentation-related complications of multilevel fusions for adult spinal deformity patients over age 65 - Surgical considerations and treatment option in patients with poor bone quality. Spine. 2006;31(19):S144-S51. doi: 10.1097/01.brs.0000236893.65878.39

17. Nachemson A. Adult scoliosis and back pain. Spine. 1979;4(6):513-7. doi: 10.1097/00007632-197911000-00011

18. Tsuchiya K, Bridwell KH, Kuklo TR, Lenke LG, Baldus C. Minimum 5-year analysis of L5-S1 fusion using sacropelvic fixation (bilateral S1 and iliac screws) for spinal deformity. Spine. 2006;31(3):303-8. doi: 10.1097/01.brs.0000197193.81296.f1

19. Kim YJ, Bridwell KH, Lenke LG, Rhim S, Cheh G. Pseudarthrosis in long adult spinal deformity instrumentation and fusion to the sacrum: Prevalence and risk factor analysis of 144 cases. Spine. 2006;31(20):2329-36. doi: 10.1097/01.brs.0000238968.82799.d9

20. Cho KJ, Suk SI, Park SR, Kim JH, Kim SS, Choi WK, et al. Complications in posterior fusion and instrumentation for degenerative lumbar scoliosis. Spine. 2007;32(20):2232-7. doi: 10.1097/BRS.0b013e31814b2d3c

21. Bridwell KH, Glassman S, Horton W, Shaffrey C, Schwab F, Zebala LP, et al. Does Treatment (Nonoperative and Operative) Improve the Two-Year Quality of Life in Patients With Adult Symptomatic Lumbar Scoliosis A Prospective Multicenter Evidence-Based Medicine Study. Spine. 2009;34(20):2171-8. doi: 10.1097/BRS.0b013e3181a8fdc8

22. Isaacs RE, Hyde J, Goodrich JA, Rodgers WB, Phillips FM. A Prospective, Nonrandomized, Multicenter Evaluation of Extreme Lateral Interbody Fusion for the Treatment of Adult Degenerative Scoliosis Perioperative Outcomes and Complications. Spine. 2010;35(26):S322-S30. doi: 10.1097/BRS.0b013e3182022e04

23. Kim YJ, Bridwell KH, Lenke LG, Glattes CR, Rhim S, Cheh G. Proximal junctional kyphosis in adult spinal deformity after segmental posterior spinal instrumentation and fusion - Minimum five-year follow-up. Spine. 2008;33(20):2179-84. doi: 10.1097/BRS.0b013e31817c0428

24. Dakwar E, Cardona RF, Smith DA, Uribe JS. Early outcomes and safety of the minimally invasive, lateral retroperitoneal transpsoas approach for adult degenerative scoliosis. Neurosurgical Focus. 2010;28(3):7. doi: 10.3171/2010.1.Focus09282

25. Smith JS, Shaffrey CI, Glassman SD, Berven SH, Schwab FJ, Hamill CL, et al. Risk-Benefit Assessment of Surgery for Adult Scoliosis An Analysis Based on Patient Age. Spine. 2011;36(10):817-24. doi: 10.1097/BRS.0b013e3181e21783

26. Silva FE, Lenke LG. Adult degenerative scoliosis: evaluation and management. Neurosurgical Focus. 2010;28(3):10. doi: 10.3171/2010.1.Focus09271

27. Yagi M, Akilah KB, Boachie-Adjei O. Incidence, Risk Factors and Classification of Proximal Junctional Kyphosis: Surgical Outcomes Review of Adult Idiopathic Scoliosis. Spine. 2011;36(1):E60-E8. doi: 10.1097/BRS.0b013e3181eeaee2

28. Terran J, Schwab F, Shaffrey CI, Smith JS, Devos P, Ames CP, et al. The SRS-Schwab Adult Spinal Deformity Classification: Assessment and Clinical Correlations Based on a Prospective Operative and Nonoperative Cohort. Neurosurgery. 2013;73(4):559-68. doi: 10.1227/neu.0000000000000012

29. Jackson RP, Peterson MD, McManus AC, Hales C. Compensatory spinopelvic balance over the hip axis and better reliability in measuring lordosis to the pelvic radius on standing lateral radiographs of adult volunteers and patients. Spine. 1998;23(16):1750-67. doi: 10.1097/00007632-199808150-00008

30. Yagi M, King AB, Boachie-Adjei O. Incidence, Risk Factors, and Natural Course of Proximal Junctional Kyphosis Surgical Outcomes Review of Adult Idiopathic Scoliosis. Minimum 5 Years of Follow-up. Spine. 2012;37(17):1479-89. doi: 10.1097/BRS.0b013e31824e4888

31. Protopsaltis T, Schwab F, Bronsard N, Smith JS, Klineberg E, Mundis G, et al. The T1 Pelvic Angle, a Novel Radiographic Measure of Global Sagittal Deformity, Accounts for Both Spinal Inclination and Pelvic Tilt and Correlates with Health-Related Quality of Life. Journal of Bone and Joint Surgery-American Volume. 2014;96A(19):1631-40. doi: 10.2106/jbjs.M.01459

32. Schwab F, Farcy JP, Bridwell K, Berven S, Glassman S, Harrast J, et al. A clinical impact classification of scoliosis in the adult. Spine. 2006;31(18):2109-14. doi: 10.1097/01.brs.0000231725.38943.ab

33. Lafage R, Schwab F, Challier V, Henry JK, Gum J, Smith J, et al. Defining Spino-Pelvic Alignment Thresholds Should Operative Goals in Adult Spinal Deformity Surgery Account for Age? Spine. 2016;41(1):62-8. doi: 10.1097/brs.0000000000001171

34. Maruo K, Ha Y, Inoue S, Samuel S, Okada E, Hu SS, et al. Predictive Factors for Proximal Junctional Kyphosis in Long Fusions to the Sacrum in Adult Spinal Deformity. Spine. 2013;38(23):E1469-E76. doi: 10.1097/BRS.0b013e3182a51d43

35. Bradford DS, Tay BKB, Hu SS. Adult scoliosis: Surgical indications, operative management, complications, and outcomes. Spine. 1999;24(24):2617-29. doi: 10.1097/00007632-199912150-00009

36. Yilgor C, Sogunmez N, Boissiere L, Yavuz Y, Obeid I, Kleinstuck F, et al. Global Alignment and Proportion (GAP) Score Development and Validation of a New Method of Analyzing Spinopelvic Alignment to Predict Mechanical Complications After Adult Spinal Deformity Surgery. Journal of Bone and Joint Surgery-American Volume. 2017;99(19):12. doi: 10.2106/jbjs.16.01594

37. Anand N, Baron EM, Thaiyananthan G, Khalsa K, Goldstein TB. Minimally Invasive Multilevel Percutaneous Correction and Fusion for Adult Lumbar Degenerative Scoliosis A Technique and Feasibility Study. Journal of Spinal Disorders & Techniques. 2008;21(7):459-67. doi: 10.1097/BSD.0b013e318167b06b

38. Smith JS, Klineberg E, Lafage V, Shaffrey CI, Schwab F, Lafage R, et al. Prospective multicenter assessment of perioperative and minimum 2-year postoperative complication rates associated with adult spinal deformity surgery. Journal of Neurosurgery-Spine. 2016;25(1):1-14. doi: 10.3171/2015.11.Spine151036

39. Pellise F, Vila-Casademunt A, Ferrer M, Domingo-Sabat M, Bago J, Perez-Grueso FJS, et al. Impact on health related quality of life of adult spinal deformity (ASD) compared with other chronic conditions. European Spine Journal. 2015;24(1):3-11. doi: 10.1007/s00586-014-3542-1

40. Smith JS, Shaffrey CI, Berven S, Glassman S, Hamill C, Horton W, et al. IMPROVEMENT OF BACK PAIN WITH OPERATIVE AND NONOPERATIVE TREATMENT IN ADULTS WITH SCOLIOSIS. Neurosurgery. 2009;65(1):86-94. doi: 10.1227/01.Neu.0000347005.35282.6c

41. Smith JS, Klineberg E, Schwab F, Shaffrey CI, Moal B, Ames CP, et al. Change in Classification Grade by the SRS-Schwab Adult Spinal Deformity Classification Predicts Impact on Health-Related Quality of Life Measures Prospective Analysis of Operative and Nonoperative Treatment. Spine. 2013;38(19):1663-71. doi: 10.1097/BRS.0b013e31829ec563

42. Bradford DS, Tribus CB. Vertebral column resection for the treatment of rigid coronal decompensation. Spine. 1997;22(14):1590-9. doi: 10.1097/00007632-199707150-00013

43. Tormenti MJ, Maserati MB, Bonfield CM, Okonkwo DO, Kanter AS. Complications and radiographic correction in adult scoliosis following combined transpsoas extreme lateral interbody fusion and posterior pedicle screw instrumentation. Neurosurgical Focus. 2010;28(3):7. doi: 10.3171/2010.1.Focus09263

44. Charosky S, Guigui P, Blamoutier A, Roussouly P, Chopin D, Study Grp S. Complications and Risk Factors of Primary Adult Scoliosis Surgery A Multicenter Study of 306 Patients. Spine. 2012;37(8):693-700. doi: 10.1097/BRS.0b013e31822ff5c1

45. Smith JS, Shaffrey CI, Berven S, Glassman S, Hamill C, Horton W, et al. Operative Versus Nonoperative Treatment of Leg Pain in Adults With Scoliosis A Retrospective Review of a Prospective Multicenter Database With Two-Year Follow-up. Spine. 2009;34(16):1693-8. doi: 10.1097/BRS.0b013e3181ac5fcd

46. Wang MY, Mummaneni PV. Minimally invasive surgery for thoracolumbar spinal deformity: initial clinical experience with clinical and radiographic outcomes. Neurosurgical Focus. 2010;28(3):8. doi: 10.3171/2010.1.Focus09286

47. Ploumis A, Transfledt EE, Denis F. Degenerative lumbar scoliosis associated with spinal stenosis. Spine Journal. 2007;7(4):428-36. doi: 10.1016/j.spinee.2006.07.015

48. Edwards CC, Bridwell KH, Patel A, Rinella AS, Berra A, Lenke LG. Long adult deformity fusions to L5 and the sacrum - A matched cohort analysis. Spine. 2004;29(18):1996-2005. doi: 10.1097/01.brs.0000138272.54896.33

49. Berven S, Deviren V, Demir-Deviren S, Hu SS, Bradford DS. Studies in the modified scoliosis research society outcomes instrument in adults: Validation, reliability, and discriminatory capacity. Spine. 2003;28(18):2164-9. doi: 10.1097/01.Brs.0000084666.53553.D6

50. Kim HJ, Bridwell KH, Lenke LG, Park MS, Song KS, Piyaskulkaew C, et al. Patients With Proximal Junctional Kyphosis Requiring Revision Surgery Have Higher Postoperative Lumbar Lordosis and Larger Sagittal Balance Corrections. Spine. 2014;39(9):E576-E80. doi: 10.1097/brs.0000000000000246

51. Mac-Thiong J-M, Transfeldt EE, Mehbod AA, Perra JH, Denis F, Garvey TA, et al. Can c7 plumbline and gravity line predict health related quality of life in adult scoliosis? Spine. 2009;34(15):E519-27. doi: 10.1097/BRS.0b013e3181a9c7ad

52. Smith JS, Shaffrey CI, Ames CP, Demakakos J, Fu KMG, Keshavarzi S, et al. Assessment of Symptomatic Rod Fracture After Posterior Instrumented Fusion for Adult Spinal Deformity. Neurosurgery. 2012;71(4):862-7. doi: 10.1227/NEU.0b013e3182672aab

53. Yadla S, Maltenfort MG, Ratliff JK, Harrop JS. Adult scoliosis surgery outcomes: a systematic review. Neurosurgical Focus. 2010;28(3):7. doi: 10.3171/2009.12.Focus09254

54. Anand N, Rosemann R, Khalsa B, Baron EM. Mid-term to long-term clinical and functional outcomes of minimally invasive correction and fusion for adults with scoliosis. Neurosurgical Focus. 2010;28(3):8. doi: 10.3171/2010.1.Focus09272

55. Bridwell KH, Lenke LG, Baldus C, Blanke K. Major intraoperative neurologic deficits in pediatric and adult spinal deformity patients. Incidence and etiology at one institution. Spine. 1998;23(3):324-31. doi: 10.1097/00007632-199802010-00008

56. Schwab F, Dubey A, Pagala M, Gamez L, Farcy JP. Adult scoliosis: A health assessment analysis by SF-36. Spine. 2003;28(6):602-6. doi: 10.1097/00007632-200303150-00016

57. Lau D, Clark AJ, Scheer JK, Daubs MD, Coe JD, Paonessa KJ, et al. Proximal Junctional Kyphosis and Failure After Spinal Deformity Surgery. Spine. 2014;39(25):2093-102. doi: 10.1097/brs.0000000000000627

58. Kim YJ, Bridwell KH, Lenke LG, Cho KJ, Enwards CC, Rinella AS. Pseudarthrosis in adult spinal deformity following multisegmental instrumentation and arthrodesis. Journal of Bone and Joint Surgery-American Volume. 2006;88A(4):721-8. doi: 10.2106/jbjs.E.00550

59. Schwab FJ, Hawkinson N, Lafage V, Smith JS, Hart R, Mundis G, et al. Risk factors for major peri-operative complications in adult spinal deformity surgery: a multi-center review of 953 consecutive patients. European Spine Journal. 2012;21(12):2603-10. doi: 10.1007/s00586-012-2370-4

60. Sansur CA, Smith JS, Coe JD, Glassman SD, Berven SH, Polly DW, et al. Scoliosis Research Society Morbidity and Mortality of Adult Scoliosis Surgery. Spine. 2011;36(9):E593-E7. doi: 10.1097/BRS.0b013e3182059bfd

61. Hostin R, McCarthy I, O'Brien M, Bess S, Line B, Boachie-Adjei O, et al. Incidence, Mode, and Location of Acute Proximal Junctional Failures After Surgical Treatment of Adult Spinal Deformity. Spine. 2013;38(12):1008-15. doi: 10.1097/BRS.0b013e318271319c

62. Cho KJ, Suk SI, Park SR, Kim JH, Kim SS, Lee TJ, et al. Short fusion versus long fusion for degenerative lumbar scoliosis. European Spine Journal. 2008;17(5):650-6. doi: 10.1007/s00586-008-0615-z

63. Lowe T, Berven SH, Schwab FJ, Bridwell KH. The SRS classification for Adult Spinal Deformity - Building on the King/Moe and Lenke Classification Systems. Spine. 2006;31(19):S119-S25. doi: 10.1097/01.brs.0000232709.48446.be

64. Smith JS, Shaffrey E, Klineberg E, Shaffrey CI, Lafage V, Schwab FJ, et al. Prospective multicenter assessment of risk factors for rod fracture following surgery for adult spinal deformity. Journal of Neurosurgery-Spine. 2014;21(6):994-1003. doi: 10.3171/2014.9.Spine131176

65. Bridwell KH, Cats-Baril W, Harrast J, Berven S, Glassman S, Farcy JP, et al. The validity of the SRS-22 instrument in an adult spinal deformity population compared with the Oswestry and SF-12 - A study of response distribution, concurrent validity, internal consistency, and reliability. Spine. 2005;30(4):455-61. doi: 10.1097/01.brs.0000153393.82368.6b

66. Bess S, Boachie-Adjei O, Burton D, Cunningham M, Shaffrey C, Shelokov A, et al. Pain and Disability Determine Treatment Modality for Older Patients With Adult Scoliosis, While Deformity Guides Treatment for Younger Patients. Spine. 2009;34(20):2186-90. doi: 10.1097/BRS.0b013e3181b05146

67. Swank S, Lonstein JE, Moe JH, Winter RB, Bradford DS. Surgical treatment of adult scoliosis. A review of two hundred and twenty-two cases. The Journal of bone and joint surgery American volume. 1981;63(2):268-87. doi: 10.2106/00004623-198163020-00013

68. Lonstein JE. Scoliosis - Surgical versus nonsurgical treatment. Clinical Orthopaedics and Related Research. 2006(443):248-59. doi: 10.1097/01.blo.0000198725.54891.73

69. Bridwell KH, Lenke LG, Cho SK, Pahys JM, Zebala LP, Dorward IG, et al. Proximal Junctional Kyphosis in Primary Adult Deformity Surgery: Evaluation of 20 Degrees as a Critical Angle. Neurosurgery. 2013;72(6):899-906. doi: 10.1227/NEU.0b013e31828bacd8

70. Pichelmann MA, Lenke LG, Bridwell KH, Good CR, O'Leary PT, Sides BA. Revision Rates Following Primary Adult Spinal Deformity Surgery Six Hundred Forty-Three Consecutive Patients Followed-up to Twenty-Two Years Postoperative. Spine. 2010;35(2):219-26. doi: 10.1097/BRS.0b013e3181c91180

71. Lafage V, Schwab F, Vira S, Patel A, Ungar B, Farcy JP. Spino-Pelvic Parameters After Surgery Can be Predicted A Preliminary Formula and Validation of Standing Alignment. Spine. 2011;36(13):1037-45. doi: 10.1097/BRS.0b013e3181eb9469

72. Cho SK, Bridwell KH, Lenke LG, Yi JS, Pahys JM, Zebala LP, et al. Major Complications in Revision Adult Deformity Surgery Risk Factors and Clinical Outcomes With 2- to 7-Year Follow-up. Spine. 2012;37(6):489-500. doi: 10.1097/BRS.0b013e3182217ab5

73. Smith JS, Lafage V, Shaffrey CI, Schwab F, Lafage R, Hostin R, et al. Outcomes of Operative and Nonoperative Treatment for Adult Spinal Deformity: A Prospective, Multicenter, Propensity-Matched Cohort Assessment With Minimum 2-Year Follow-up. Neurosurgery. 2016;78(6):851-61. doi: 10.1227/neu.0000000000001116

74. Youssef JA, Orndorff DO, Patty CA, Scott MA, Price HL, Hamlin LF, et al. Current status of adult spinal deformity. Global spine journal. 2013;3(1):51-62. doi: 10.1055/s-0032-1326950

75. Yagi M, Rahm M, Gaines R, Maziad A, Ross T, Kim HJ, et al. Characterization and Surgical Outcomes of Proximal Junctional Failure in Surgically Treated Patients With Adult Spinal Deformity. Spine. 2014;39(10):E607-E14. doi: 10.1097/brs.0000000000000266

76. Kostuik JP, Hall BB. Spinal fusions to the sacrum in adults with scoliosis. Spine. 1983;8(5):489-500. doi: 10.1097/00007632-198307000-00006

77. Bridwell KH, Baldus C, Berven S, Edwards C, Glassman S, Hamill C, et al. Changes in Radiographic and Clinical Outcomes With Primary Treatment Adult Spinal Deformity Surgeries From Two Years to Three-to Five-Years Follow-up. Spine. 2010;35(20):1849-54. doi: 10.1097/BRS.0b013e3181efa06a

78. Smith JS, Shaffrey CI, Lafage V, Blondel B, Schwab F, Hostin R, et al. Spontaneous improvement of cervical alignment after correction of global sagittal balance following pedicle subtraction osteotomy Presented at the 2012 Joint Spine Section Meeting Clinical article. Journal of Neurosurgery-Spine. 2012;17(4):300-7. doi: 10.3171/2012.6.Spine1250

79. Cho KJ, Suk SI, Park SR, Kim JH, Kang SB, Kim HS, et al. Risk Factors of Sagittal Decompensation After Long Posterior Instrumentation and Fusion for Degenerative Lumbar Scoliosis. Spine. 2010;35(17):1595-601. doi: 10.1097/BRS.0b013e3181bdad89

80. Kobayashi T, Atsuta Y, Takemitsu M, Matsuno T, Takeda N. A prospective study of de novo scoliosis in a community based cohort. Spine. 2006;31(2):178-82. doi: 10.1097/01.brs.0000194777.87055.1b

81. Grubb SA, Lipscomb HJ, Coonrad RW. Degenerative adult onset scoliosis. Spine. 1988;13(3):241-5. doi: 10.1097/00007632-198803000-00004

82. Soroceanu A, Burton DC, Oren JH, Smith JS, Hostin R, Shaffrey CI, et al. Medical Complications After Adult Spinal Deformity Surgery Incidence, Risk Factors, and Clinical Impact. Spine. 2016;41(22):1718-23. doi: 10.1097/brs.0000000000001636

83. Daffner SD, Vaccaro AR. Adult degenerative lumbar scoliosis. American journal of orthopedics (Belle Mead, NJ). 2003;32(2):77-82. doi:

84. Tribus CB. Degenerative lumbar scoliosis: evaluation and management. The Journal of the American Academy of Orthopaedic Surgeons. 2003;11(3):174-83. doi:

85. Grubb SA, Lipscomb HJ, Suh PB. Results of surgical treatment of painful adult scoliosis. Spine. 1994;19(14):1619-27. doi: 10.1097/00007632-199407001-00011

86. Blondel B, Schwab F, Ungar B, Smith J, Bridwell K, Glassman S, et al. Impact of Magnitude and Percentage of Global Sagittal Plane Correction on Health-Related Quality of Life at 2-Years Follow-Up. Neurosurgery. 2012;71(2):341-8. doi: 10.1227/NEU.0b013e31825d20c0

87. Leven DM, Lee NJ, Kothari P, Steinberger J, Guzman J, Skovrlj B, et al. Frailty Index Is a Significant Predictor of Complications and Mortality After Surgery for Adult Spinal Deformity. Spine. 2016;41(23):E1394-E401. doi: 10.1097/brs.0000000000001886

88. Bess S, Line B, Fu KM, McCarthy I, Lafage V, Schwab F, et al. The Health Impact of Symptomatic Adult Spinal Deformity: Comparison of Deformity Types to United States Population Norms and Chronic Diseases. Spine. 2016;41(3):224-33. doi: 10.1097/brs.0000000000001202

89. Phillips FM, Isaacs RE, Rodgers WB, Khajavi K, Tohmeh AG, Deviren V, et al. Adult Degenerative Scoliosis Treated With XLIF Clinical and Radiographical Results of a Prospective Multicenter Study With 24-Month Follow-up. Spine. 2013;38(21):1853-61. doi: 10.1097/BRS.0b013e3182a43f0b

90. Birknes JK, White AP, Albert TJ, Shaffrey CI, Harrop JS. Adult degenerative scoliosis: A review. Neurosurgery. 2008;63(3):A94-A103. doi: 10.1227/01.Neu.0000325485.49323.B2

91. Ploumis A, Liu H, Mehbod AA, Transfeldt EE, Winter RB. A Correlation of Radiographic and Functional Measurements in Adult Degenerative Scoliosis. Spine. 2009;34(15):1581-4. doi: 10.1097/BRS.0b013e31819c94cc

92. Mok JM, Cloyd JM, Bradford DS, Hu SS, Deviren V, Smith JA, et al. Reoperation After Primary Fusion for Adult Spinal Deformity Rate, Reason, and Timing. Spine. 2009;34(8):832-9. doi: 10.1097/BRS.0b013e31819f2080

93. Kebaish KM, Neubauer PR, Voros GD, Khoshnevisan MA, Skolasky RL. Scoliosis in Adults Aged Forty Years and Older Prevalence and Relationship to Age, Race, and Gender. Spine. 2011;36(9):731-6. doi: 10.1097/BRS.0b013e3181e9f120

94. Bridwell KH, Berven S, Glassman S, Hamill C, Horton WC, Lenke LG, et al. Is the SRS-22 instrument responsive to change in adult scoliosis patients having primary spinal deformity surgery? Spine. 2007;32(20):2220-5. doi: 10.1097/BRS.0b013e31814cf120

95. Baron EM, Albert TJ. Medical complications of surgical treatment of adult spinal deformity and how to avoid them. Spine. 2006;31(19):S106-S18. doi: 10.1097/01.brs.0000232713.69342.df

96. Enercan M, Ozturk C, Kahraman S, Sarier M, Hamzaoglu A, Alanay A. Osteotomies/spinal column resections in adult deformity. European Spine Journal. 2013;22:S254-S64. doi: 10.1007/s00586-012-2313-0

97. Albert TJ, Purtill J, Mesa J, McIntosh T, Balderston RA. Health outcome assessment before and after adult deformity surgery. A prospective study. Spine. 1995;20(18):2002-4; discussion p5. doi: 10.1097/00007632-199509150-00009

98. Schwab FJ, Lafage V, Farcy JP, Bridwell KH, Glassman S, Shainline MR. Predicting outcome and complications in the surgical treatment of adult scoliosis. Spine. 2008;33(20):2243-7. doi: 10.1097/BRS.0b013e31817d1d4e

99. Edwards CC, Bridwell KH, Patel A, Rinella AS, Kim YJ, Berra A, et al. Thoracolumbar deformity arthrodesis to L5 in adults: The fate of the L5-S1 disc. Spine. 2003;28(18):2122-31. doi: 10.1097/01.Brs.0000084266.37210.85

100. Kim HJ, Bridwell KH, Lenke LG, Park MS, Ahmad A, Song KS, et al. Proximal Junctional Kyphosis Results in Inferior SRS Pain Subscores in Adult Deformity Patients. Spine. 2013;38(11):896-901. doi: 10.1097/BRS.0b013e3182815b42
